# An L-Slot Frequency Reconfigurable Antenna Based on MEMS Technology

**DOI:** 10.3390/mi14101945

**Published:** 2023-10-18

**Authors:** Yu Chen, Honglei Guo, Yanfei Liu, Jing Li, Yongxin Zhan, Qiannan Wu, Mengwei Li

**Affiliations:** 1 School of Instrument and Electronics, North University of China, Taiyuan 030051, China; cy13833876975@163.com (Y.C.); sz202106060@st.nuc.edu.cn (H.G.); lijing@tit.edu.cn (J.L.); z525649725@163.com (Y.Z.); 2 The Academy for Advanced Interdisciplinary Research, North University of China, Taiyuan 030051, China; 15373166906@163.com; 3 The Center for Microsystem Integration, North University of China, Taiyuan 030051, China; 4 School of Semiconductor and Physics, North University of China, Taiyuan 030051, China

**Keywords:** frequency reconfigurable, high-frequency band, patch antenna, RF MEMS switch

## Abstract

Given the shortage of spectrum resources and the demand for communication systems of diminutive size, multi-function, and adaptive characteristics, this paper proposes an L-slot frequency reconfigurable antenna based on the MEMS switch. The antenna size is 4.07 × 5.27 mm^2^ and is suitable for the U-band. The antenna structure consists of two RF MEMS switches, a Rogers RT5880 dielectric substrate, an L-slot patch, and a full-coverage ground. The switch is of a series contact structure and is arranged at the corner of an L-slot. By controlling the on and off state of the switch, the antenna can switch between four states of 42.36, 47.65, 53.13, and 56.72 GHz. According to the simulation results in CST STUDIO SUITE 2018, the maximum gain of the antenna is 7.90 dB, the impedance bandwidth of each state is above 1 GHz, and the direction is mainly consistent. The antenna can meet the demand for multi-frequency millimeter wave communication.

## 1. Introduction

Advances in wireless communication technology have led to a shortage of spectrum resources, and the restriction of spectrum resources has led to the development of multi-standard, multi-functional devices. To improve spectrum utilization, cognitive radio (CR) networks use spectrum awareness to detect currently unused frequency bands and adjust their parameters to accommodate free resources to enable normal communication [1]. As a key component in wireless communication systems for sending and receiving electromagnetic waves, antennas are pre-emptively affected. At this stage, solutions in this phase are mainly ultra-broadband, tunable, with high transmission capacity and efficiency, such as broadband antennas, reconfigurable antennas, array antennas, MIMO antennas, etc. Among them, the reconfigurable antenna can dynamically adjust its operating frequency, radiation pattern, and gain according to changes in communication requirements and environment. The adaptive nature of reconfigurable antennas can not only improve the current situation of scarce spectrum resources, but also replace multiple antennas with fixed parameters to reduce the system size and complexity. In addition, it can suppress interference signals in complex radio environments to ensure communication quality. Compared to the limitations of broadband antennas in terms of size, weight, and radiation efficiency, as well as the disadvantages of complex structures and the difficult calibration and maintenance of array antennas, reconfigurable antennas show unique advantages. It can be used in a wide range of systems, such as wireless communications, smart transportation, radar systems, drone swarms, and electronic countermeasures.

The key technologies in 5G are wideband and high-frequency transmission. The main operating bands of conventional mobile communications are concentrated below 3 GHz, and the spectrum resources are tight. However, the available resources at high frequencies are abundant and can even occupy a wider continuous operating band to meet the future demand for channel capacity and transmission rate [2]. Because of the ultra-short wavelength of millimeter waves, millimeter wave devices naturally have the advantages of small size and high integration. As a result, multiple antennas can be centrally arranged in a smaller area, which can satisfy both isolation and multi-frequency coverage and high directivity beam allocation techniques.

The search for methods to implement high-frequency reconfigurable antennas has become a hot research topic based on current application requirements. Depending on the operating characteristics and application scenarios, reconfigurable antennas can be classified as frequency reconfigurable, directional map reconfigurable, polarization reconfigurable, and hybrid reconfigurable [3]. The reconfigurable implementation methods include using liquid metal [4], carrying tunable material [5], changing the mechanical structure of the antenna, and loading the switch for electrical adjustments [6,7,8]. Among them, the loading switch is simple and flexible and can make the antenna structure relatively compact. The commonly applied switches are PIN diodes, varactor diodes, MEMS switches, etc. [9,10,11]. For example, in 2007, Kagan Topalli introduced a dual-band reconfigurable gap dipole antenna array controlled by an MEMS switch, capable of achieving peak gains of 7.4 dBi and 11.1 dBi at 10 GHz and 16 GHz, respectively [12]. In 2013, a U-shaped slotted antenna using three RF MEMS switches was proposed to achieve the reconfigurable characteristics of the antenna [13]. In 2018, a frequency reconfigurable antenna based on an MEMS switch designed by the North University of China could work in four different frequency states, covering 14 GHz to 22 GHz [14]. In 2020, Hassan et al. proposed a novel two-element multiple input multiple outputs (MIMO) reconfigurable antenna that can be switched among 600 MHz, 1.8, 2.4, 3.5, and 5.5 GHz bands [15]. In 2020, a novel design of a compact low-cost quad band reconfigurable antenna is presented by Singh et al. It achieves four frequency states (9.92/14.86/18.21/21.2 GHz) with four PIN switches and has a dimension of 30 × 36.7 mm^3^ [16]. In 2021, Song et al. proposed the introduction of on-chip switches to realize a frequency reconfigurable antenna in order to enhance the bandwidth (29.5~51.0 GHz) of the antenna [17]. However, the gain is only 3.3 dBi. However, the aforementioned reconfigurable antennas have a larger size, a lower operating band, fewer tunable modes, and a narrower range of tunable frequencies, which still do not meet the future requirements of millimeter wave wireless communications.

In this paper, we propose an MEMS switch-based L-slot frequency reconfigurable antenna for the U-band, capable of four tuning states. The RF MEMS switch applied in this antenna has desirable switching characteristics, such as excellent linearity, high isolation, low insertion loss, and miniaturization. The current distribution around the L-slot is tuned by controlling the on-off state of the MEMS switch, which modifies the effective electrical length of the antenna and enables frequency reconfiguration. It can be applied to 5G millimeter wave communication, unmanned driving, and telemetry.

## 2. Theory and Design

### 2.1. Design of the RF MEMS Switch

In high-frequency communications, the low insertion loss of RF MEMS switches helps to maintain the RF signal quality, high isolation can reduce crosstalk between signals, high linearity can maintain signal integrity, and small size facilitates integration. It has outstanding benefits. In addition, the processing techniques for RF MEMS switches are relatively mature [18], while the integration with the antenna is relatively high in terms of the manufacturing process. It shows great potential in designing millimeter wave reconfigurable antennas [19]. RF MEMS switches are therefore used to electronically control the antenna frequency in this paper.

**Figure 1:**
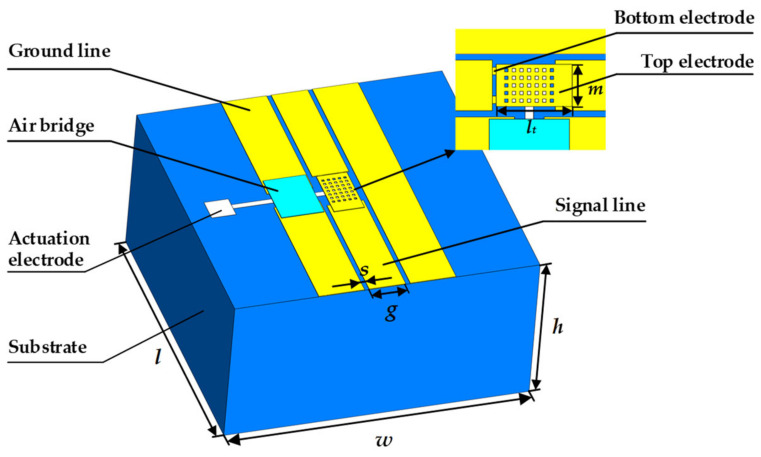
Schematic of the RF MEMS switch structure.

The RF MEMS switch designed in this paper consists of a borosilicate glass substrate, a straight-plate cantilever-beam top electrode, an actuation electrode, coplanar waveguide (CPW) lines, and an air bridge, as shown in Figure 1. Details of the switch sizes are given in Table 1. The actuation electrode is positioned below the top electrode and activates the switch by electrostatic actuation. A voltage is applied to the actuation electrode to operate the switch. When the top electrode is pulled down to (2/3)*g*_0_ by electrostatic force, the increase in electrostatic force is much greater than the increase in restoring force, resulting in a rapid drop-down of the top electrode [20]. At this point, the top electrode contacts the bottom electrode, and the signal is transmitted. After removing the voltage, the switch returns to its original state, and the signal is then disconnected. The voltage that pulls the top electrode down to (2/3)*g*_0_ is the driving voltage. Its expression is:

(1) $$V=\sqrt{\frac{8k}{27\varepsilon_{0}A}g_{0}^{3}}$$

where *ε*_0_ is the dielectric constant of the vacuum. *g*_0_ is the distance between the top electrode and the driving electrode, which is 3 μm. *A* is the opposite area between the top electrode and the driving electrode, which is 90 × 100 μm^2^. In addition, *k* is the equivalent elasticity coefficient of the top electrode. *k* is calculated as follows:

(2) $$k=\frac{1}{4}Em{\left(\frac{h_{t}}{l_{t}}\right)}^{3}$$

where *E* is the Young’s modulus of the cantilever material, *m* is the width of the top electrode, *l_t_* is the length of the top electrode, and *h_t_* is the thickness of the top electrode. The equivalent elasticity coefficient *k* was calculated as 1.9875 N/m. Therefore, the theoretical value of the switch driving voltage from the above equations is 14 V.

**Figure 2:**
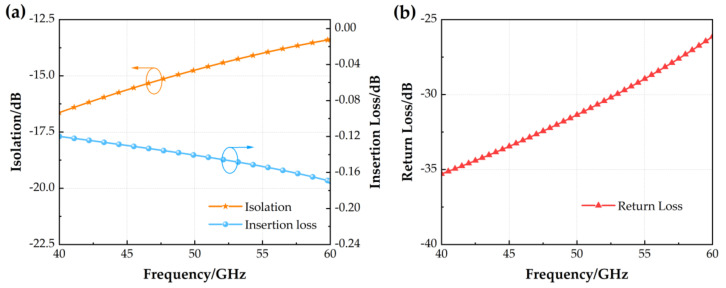
Parameters of S for the RF MEMS switch (**a**) Isolation and insertion loss; (**b**) Return loss.

The simulation results of the S-parameters of the MEMS switch, including isolation and insertion loss, are shown in Figure 2. When the switch is on, the insertion loss value is ≤0.17 dB and the return loss value is ≥26 dB in the 40~60 GHz band. When the switch is off, the isolation is ≥15 dB in the 40~48.5 GHz band and ≥13.4 dB in the 48.5~60 GHz band. As can be seen, the MEMS switch has better RF performance in high-frequency bands and can support the design of additional reconfigurable antennas.

### 2.2. Design of a Frequency Reconfigurable Antenna

**Figure 3:**
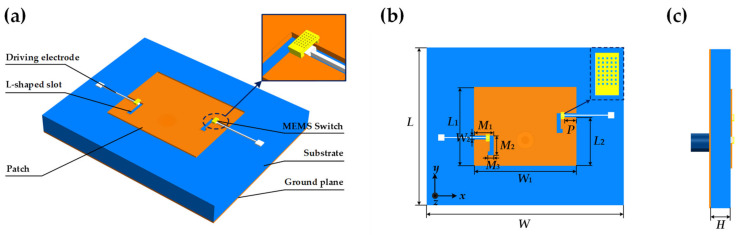
L-slot frequency reconfigurable antenna (**a**) Stereogram; (**b**) Front view; (**c**) Side view.

The structure of the coaxial fed L-slot frequency reconfigurable antenna based on RF MEMS switches is shown in Figure 3. The antenna is designed on a Rogers RT5880 substrate with a thickness of 0.254 mm (*ε_r_* = 2.2). The radiation patch is located on the upper surface of the substrate. An L-slot is opened on the left and right sides of the patch, and a straight plate-type MEMS switch is loaded at the corner of the slot. Driving electrodes located on the same side apply an on-state voltage to the switch, and the frequency tunable function is implemented by controlling the on-off of the MEMS switch. The ground plane of the antenna is located on the lower surface of the substrate and uniformly covers the entire plane. The detailed dimensions of the antennas are given in Table 2.

**Figure 4:**
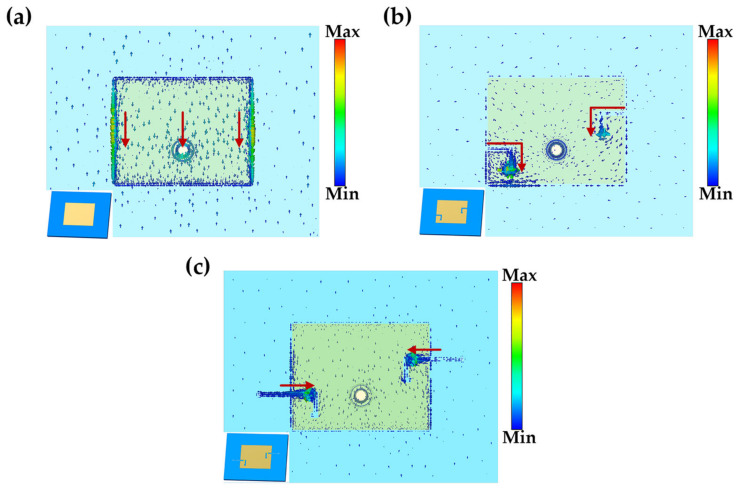
The current distribution on the antenna surface is shown in the figure, and the red arrows indicate the direction in which the currents converge. (**a**) The state without slot and switch; (**b**) The state with slot but no switch; (**c**) The state with slot and switch.

On the one hand, the L-slot changes the current path on the surface of the radiation patch, and on the other hand, the addition of the RF MEMS switch further controls the current density at the slot. Both of them together modify the radiation properties of the antenna to enable four-frequency state switching. For the convenience of illustrating the working principle of the reconfigurable antenna, the surface current distributions of the antenna in the states without slot and switch, with slot but no switch, and with slot and switch are listed below, as shown in Figure 4. Figure shows that the current clusters flow around the edges of the patch in the absence of gaps and without switching and are denser on the left and right sides. The large amount of current inside the patch is uniformly shifted up and down. The current is transferred along the slot shape when there is a slot but no switching state, and then collects in large quantities at the end of the gap. With the slot and switch state (this state sets the switch to be entirely on), current flows along the slot inlet to the switch. A large amount of current is concentrated on the switch. Only a finite fraction of the current flows toward the end of the gap.

## 3. Simulation Result

In this paper, we use CST STUDIO SUITE 2018 electromagnetic simulation software to build the base model of the antenna. Then, build the frequency reconfigurable antenna model by optimizing the length and shape of the slot and the position of the RF MEMS switches. The excitation is applied to the antenna at the coaxial feed to simulate the shift of the reflection coefficient, orientation map, and gain of the antenna in the 40~60 GHz band in different states.

Step 1: Establish a basic model of the antenna without slots and switches. The size of the patch is determined according to the relative permittivity (Rogers RT5880, *ε_r_* = 2.2), thickness (*H* = 0.254 mm) and antenna operating frequency (*f_r_* > 40 GHz) of the selected dielectric substrate. Based on the following formula [21,22], the width of the rectangular patch is:

(3) $$W=\frac{c}{2f_{r}}{\left(\frac{\varepsilon_{r}+1}{2}\right)}^{-1/2}$$

When *W*/*H* is much greater than 1, the expression of the equivalent permittivity is

(4) $$\varepsilon_{e}=\frac{\varepsilon_{r}+1}{2}+\frac{\varepsilon_{r}-1}{2}{\left(1+\frac{12H}{W}\right)}^{-1/2}$$

Due to the edge effect, each side of the patch is extended by Δl in the direction parallel to *L*. *L* is modified by *ε*_e_ and *W*/*H*. Common expressions are

(5) $$\Delta l=0.412H\frac{\left(\varepsilon_{e}+0.3\right)\left(W/H+0.264\right)}{\left(\varepsilon_{e}-0.258\right)\left(W/H+0.8\right)}$$

The equivalent length of the patch is

(6) $$L=\frac{c}{2f_{r}\sqrt{\varepsilon_{e}}}-2\Delta l$$

In Equations (3)–(6), *W* represents the patch width, *L* represents the patch length, c is the speed of light, *ε_r_* and *ε_e_* represent the relative permittivity and the equivalent permittivity of the dielectric substrate, and *f_r_* represents the resonant frequency of the antenna.

**Figure 5:**
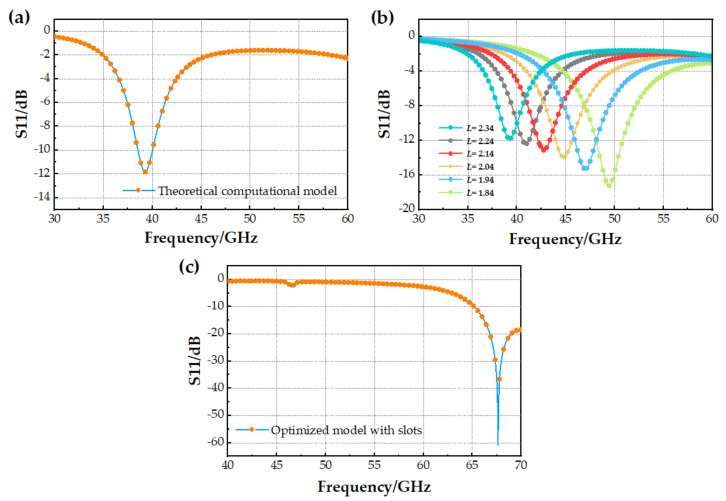
S11 results during antenna model optimization. (**a**) Theoretical computational model; (**b**) Patch width optimization process; (**c**) Optimized model with slots.

According to the formula above, the size of the proposed patch is as follows: *W* = 2.68 mm and *L* = 2.34 mm. The simulation results show that the resonant frequency of the antenna is 39.24 GHz, and the value of S11 is smaller, as shown in Figure 5a. After optimization, the operating frequency of the antenna is increased by reducing the length *L* of the patch, while the size of the substrate remains unchanged. The optimization process is shown in Figure 5b. As can be seen from Figure 5b, decreasing *L* causes the frequency to shift to the right, and the value of S11 to increase. Therefore, *L* is set to 2.04 mm in this paper, which is the choice when considering the operating frequency and the overall matching effect of the antenna. The operating frequency for this size is 43.47~46.21 GHz.

Step 2: Build an antenna model with slots but no switches. Based on Step 1, add the L-slot on the left and right sides of the patch, respectively. At this point, the resonant frequency of the antenna is shifted from 44.8 GHz to 67.63 GHz, with an offset reaching 22.83 GHz. The S11 curve is shown in Figure 5c. It can be predicted that after adding the MEMS switch on the L-slot, the resonant frequency of the antenna will float between the resonant frequencies of the antennas in Step 1 and Step 2.

**Figure 6:**
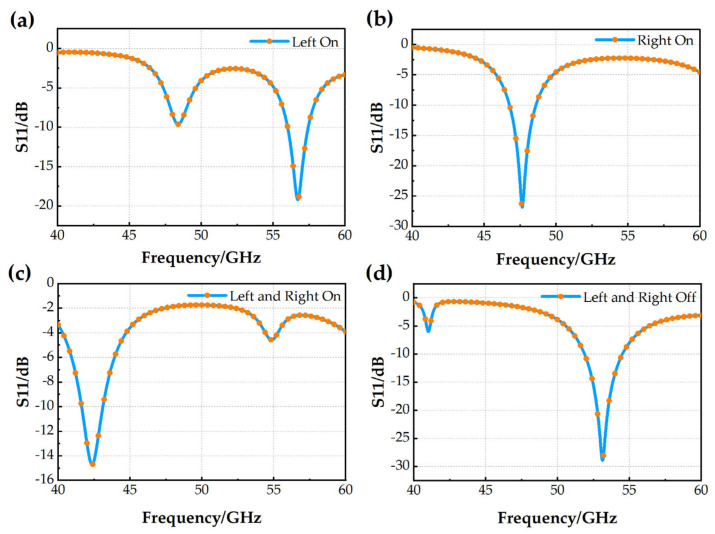
S11 results for the antenna model with slots and switches after Step 3: (**a**) state 1; (**b**) state 2; (**c**) state 3; (**d**) state 4.

Step 3: Build the antenna model with slots and switches. Based on the model in Step 2, the MEMS switch is added at the corner of the L-slot. The activated/deactivated state of the switch is controlled by applying and removing the voltage to the drive electrode. The S11 curve of the antenna when the state of the switch is tuned is shown in Figure 6. The left switch is on as state 1, the right switch is on as state 2, both left and right switches are on as state 3, and both left and right switches are off as state 4. The −10 dB impedance bandwidths for the four states are 1.43 GHz (56.01~57.44 GHz), 1.84 GHz (46.76~48.60 GHz), 1.48 GHz (41.63~43.11 GHz), and 2.63 GHz (51.88~54.51 GHz). The impedance bandwidth of the antenna in all four states is above 1 GHz, with a minimum S11 value of 14.7 dB and a maximum of 28.9 dB.

**Figure 7:**
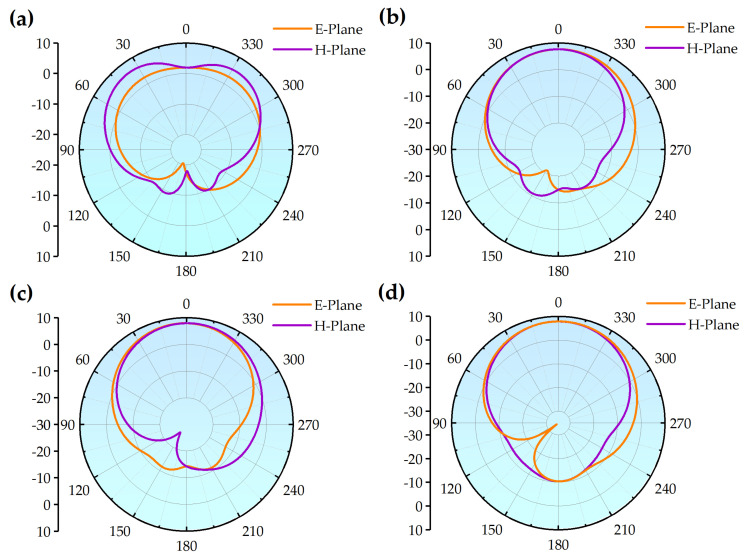
Antenna far-field radiation direction diagram (**a**) state 1; (**b**) state 2; (**c**) state 3; (**d**) state 4.

Thus far, the L-slot frequency reconfigurable antenna model based on RF MEMS switches has been obtained after three optimization steps. Figure 7 shows the far-field radiation direction maps of the antennas in the E-plane and the H-plane at the four operating frequency points. As can be seen in Figure, the antenna orientation is fundamentally the same across the four states, exhibiting stable directional radiation properties. Figure 7a shows the radiation direction diagram of the antenna working at 56.72 GHz when the left switch is on. Although its H-plane curve is slightly distorted compared to the other frequency points, the main radiation direction range is almost the same, within acceptable limits.

**Figure 8:**
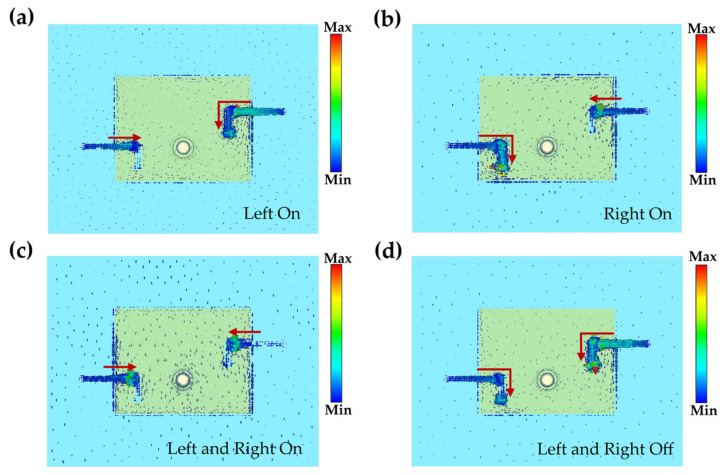
The current distribution diagram of the antenna in different operating modes is shown in the figure, and the red arrows indicate the direction in which the currents converge. (**a**) state 1; (**b**) state 2; (**c**) state 3; (**d**) state 4.

In this paper, the current distribution of the antenna in the four modes is shown in Figure 8. It can be found that when the switch is on, the current is mainly concentrated near the switch. When the switch is switched off, the current rushes along the L-slot to the end. The difference in the current distribution of the antenna in the four states is significant enough to conclude that RF MEMS switches are the key factor affecting the frequency variation. There is inevitable current convergence due to the presence of the driving electrodes.

**Figure 9:**
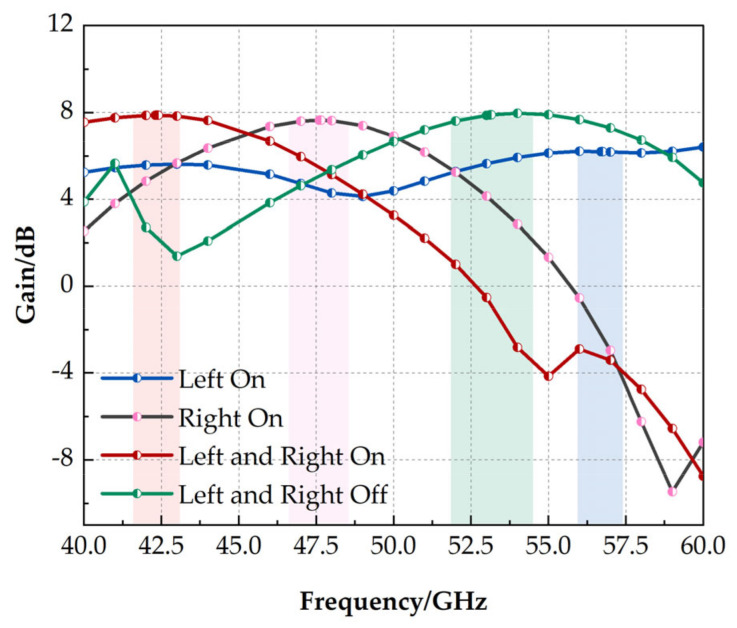
Antenna radiation gain.

The gain of the antenna in different operating modes is shown in Figure 9. The maximum gain of the antennas in the four operating states is 7.88 dB, 7.60 dB, 7.90 dB, and 6.20 dB. Overall, the gain of the antenna is considerable.

### 3.1. Effects of Structural Parameters on the Antenna

The size and shape of the slot, as well as the position of the RF MEMS switch, directly affect the resonant frequency and radiation performance of the antenna. Since the electric field direction is parallel to the YOZ plane, the slot along the *X*-axis has a larger effect on the current cutoff. Thus, the starting end of the slot is cut in the direction along the *X*-axis. The second section of the slot, which turns into the *Y*-axis direction, has less cutting effect on the current and belongs to the transition region. Finally, the end fold-back part of the L-slot changes the current path a second time. The on-off state of the RF MEMS switch and its layout are then used to achieve frequency tunability.

**Figure 10:**
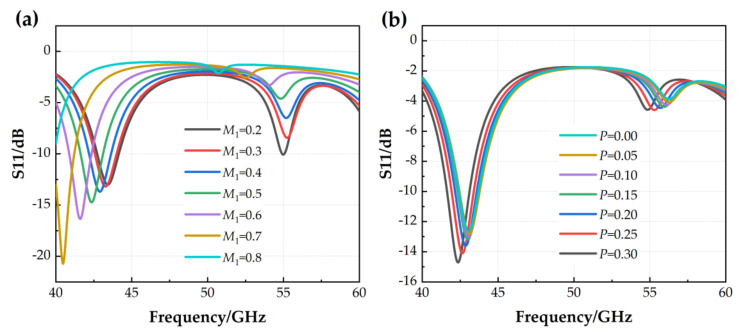
Parameter optimization: (**a**) The effect of *M*_1_ on the S11 parameters; (**b**) The effect of *P* on the S11 parameters.

In the following, state 4 (both left and right are disconnected) is taken as an example to further illustrate the effects of slot size and switch position on antenna performance, according to the parameter optimization and analysis process. First, Figure 10a shows the effect of the length of the cut-in section of the L-slot along the *Y*-axis on antenna S11 parameters. It can be seen that the modal value of the reflection coefficient gradually increases with *M*_1_, and the resonant frequency shifts to lower frequency bands. This indicates that the longer the slot along the *X*-axis, the longer the electrical length of the current flowing through it, and thus the antenna can operate at lower frequencies. Additionally, Figure 10b shows the effect of the switch position on the antenna reflection coefficient. *P* indicates the distance from the RF MEMS switch to the edge of the patch. As the value of *P* increases, the modulus of the reflection coefficient increases, and the resonance point shifts to the left. Again, the closer the switch position is to the center, the greater the effect on the path of the current, making the resonance frequency lower. In this paper, we choose *M*_1_ = 0.5 mm and *P* = 0.3 mm considering the size factor and parameter index of the antenna.

### 3.2. Comparison and Discussion

Table 3 provides a brief comparison between the L-slot MEMS frequency reconfigurable antenna proposed in this paper and previous antennas of the same type [15,16,17,23]. The antenna designed in this paper has a small size, high frequency, and a large gain. With the same number of switches, it has more tunable states.

## 4. Process Scheme and Measurement

### 4.1. Process Program

**Figure 11:**
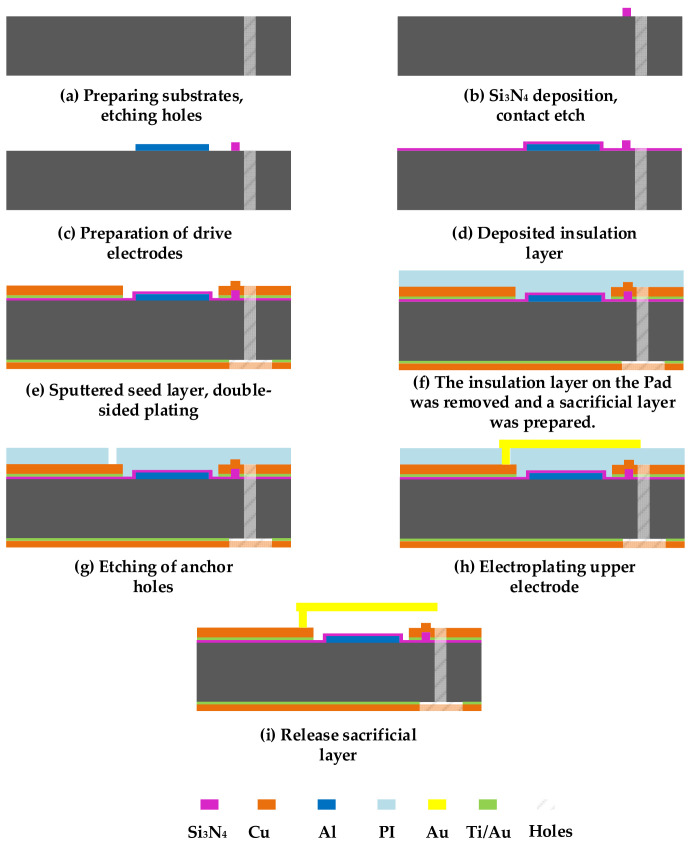
Process program for L-slot frequency reconfigurable antenna.

According to the antenna structure, a technological preparation scheme is developed. The implementation steps are as follows: The first step is to prepare the substrate and etch the holes. In this paper, Rogers RT5880 is selected as the substrate material and is deep etched using APEX SLR ICP2 equipment to a depth of 254 μm as shown in Figure 11a. The holes are internal hidden structures that are not visible within the profile. In step 2, a 400 nm thick layer of Si_3_N_4_ is prepared by plasma-enhanced chemical vapor deposition (PECVD) and etched to form the switch contacts, as described in Figure 11b. In step 3, a 500 nm aluminum (Al) layer is grown by magnetron sputtering and etched in phosphoric acid solution to form the switching driver electrode, as shown in Figure 11c. In step 4, a layer of Si_3_N_4_ with a thickness of 400 nm is deposited on the driver electrode by PECVD to prevent crosstalk among the signals, as shown in Figure 11d. In step 5, a Ti/Cu seed layer is sputtered onto the substrate, and the top patch structure and bottom ground layer are simultaneously prepared by water bath plating. The seed layer is then removed by wet etching, as shown in Figure 11e. In step 6, the silicon nitride isolation layer on the pad is removed by photolithography, and then the polyimide sacrificial layer is prepared by the rotary coating method, as can be seen in Figure 11f. In step 7, as shown in Figure 11g, the anchor holes in the sacrificial layer are created by wet etching. In step 8, the top electrode of the RF MEMS switch is formed by sputtering a gold seed layer and plating a 2 µm thick gold layer. The seed layer is removed as shown in Figure 11h. In step 9, the L-slot frequency reconfigurable antenna is finally obtained by releasing the PI sacrifice layer by reactive ion etching (RIE) in Figure 11i.

### 4.2. Experimental Validation

**Figure 12:**
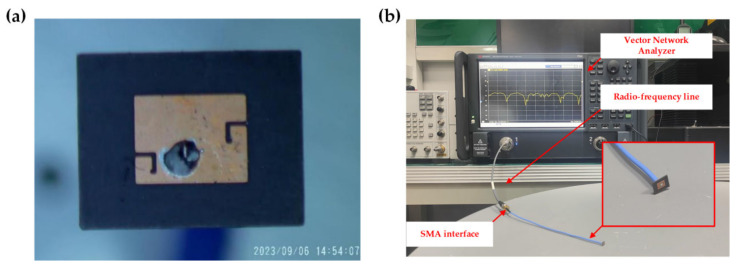
Measurement (**a**) A prototype of the antenna as a state without switches; (**b**) Microwave performance test of the antenna.

**Figure 13:**
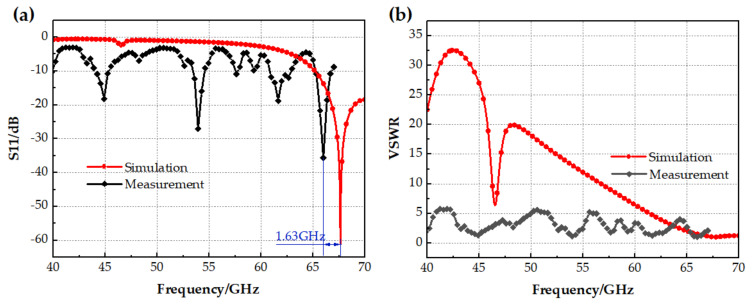
Experimental verification of the state without switches (**a**) The comparison of the S11 parameter between the simulation and measurement; (**b**) The comparison of the VSWR parameter between the simulation and measurement.

Due to uncontrollable factors of the preparation platform and the preparation cycle, only the prototype without the switch is presented in this paper, as shown in Figure 12a. Figure 12b shows the diagram of the field test. The main instruments used were a vector network analyzer (the frequency range of this vector network analyzer is DC~67 GHz) and an RF line. Figure 13 shows the comparison of parameters between the simulation and measurement of the no-switch condition. As shown in Figure 13a, the test result of S11 is shifted 1.63 GHz to the left of the simulation result, and the −10 dB operating bandwidth is 1.5 GHz. There are also additional resonance points in the 40 to 70 GHz range, including 44.9 GHz, 54 GHz, and 61.6 GHz. Figure 13b shows that the deviation of the VSWR is large in the range of 40 GHz to 50 GHz. The main reasons for the above deviations are errors in the preparation process, interference in the test conditions, and systematic errors. First, since the size of the antenna is in the order of millimeters, the solder joints at the feed ports will have some effect on the structure and flatness of the antenna radiating unit [24], as clearly shown in Figure 12a. This is the likely cause of the multiple resonance points and the leftward shift in frequency. The second reason is the limitation of experimental conditions; the measurements were not completed in the microwave darkroom, so the electromagnetic environment affected the antenna radiation [25]. In addition, bending and vibration of the test cable and torsion of the SMA connector increase the uncertainty of the test, resulting in systematic errors that affect measurement accuracy [26]. Overall, however, the errors in the key frequency range of 65~67 GHz are acceptable.

## 5. Conclusions

In this paper, based on the development of communication technologies, we temporarily analyze the utility value of future reconfigurable antennas and propose an L-slot MEMS frequency reconfigurable antenna for the U-band. The reconfigurable function of the antenna is performed by RF MEMS switches. The L-slot widens the frequency range and creates reconfigurable conditions for it. Two switches with four matching states enabled the antenna to achieve better impedance matching at 42.36, 47.65, 53.13, and 56.72 GHz. The results show that the proposed L-slot MEMS frequency reconfigurable antenna is one of the ideal choices for future millimeter wave communication systems. In addition, a process preparation scheme is proposed in this paper. However, due to the slow progress of the work, only the state without the switch is tested. The test results are within acceptable limits. Preparation and testing will continue in the future.

## Figures and Tables

**Table 1:** Structural parameters of the RF MEMS switch.

Dimension	Value (μm)	Comments
*g*	120	Width of signal line
*s*	14.7	Distance between signal and ground lines
*m*	100	Width of top electrode
*l_t_*	180	Length of top electrode
*h*	500	Thickness of substrate
*w*	1000	Width of substrate
*l*	1000	Length of substrate

**Table 2:** Structural Parameters of L-slot Frequency Reconfigurable Antenna.

Dimension	Value (mm)	Comments
*W*	5.27	Width of antenna
*L*	4.07	Length of antenna
*W*_1_	2.68	Width of radiation patch
*L*_1_	2.04	Length of radiation patch
*W*_2_	0.10	Width of L-slot
*M*_1_	0.50	Length of L-slot along x-direction
*M*_2_	0.50	Length of L-slot along y-direction
*M*_3_	0.15	Length of L-slot folding back part
*L*_2_	1.31	Distance of L-slot and radiation patch edges.
*P*	0.30	Distance of RF MEMS switch and radiation patch edges.
*H*	0.254	Thickness of substrate

**Table 3:** Comparison of Frequency Reconfigurable Antenna.

Ref.	Size (mm^3^)	Number of Switches	Resonant Frequency *f*_0_ (GHz)	Bandwidth (GHz)	Adjustable Status	Maximum Gain (dB)
[15]	10 × 10 × 0.5	2	14.25/14.5/16.3/16.4/16.65 /18.8/20.8	14~15.2/15.8~17.3/18.6~19.1/20.6~21	4	-
[16]	30 × 36.7	4	9.92/14.86/18.21/21.2	9.5~10.3/14.3~15.2/17.8~19/20.5~22	4	-
[17]	1.1 × 1.7	2	40	30~52.5	2	3.3
[23]	31 × 42.04	2	3.6/3.1/10/10/10.2/11.3/11.4 /11.41/14/14.1/14.11	-	3	2.27
This work *	4.07 × 5.27 × 0.254	2	42.36/47.65/53.13/56.72	41.63~43.11/46.76~48.60/51.88~54.51 /56.01~57.44	4	7.90

* The data shown are simulated only.

## Data Availability

The data that support the findings of this study are available from the corresponding author upon reasonable request.
